# Neoadjuvant therapy in pancreatic cancer: what is the true oncological benefit?

**DOI:** 10.1007/s00423-020-01946-4

**Published:** 2020-08-10

**Authors:** Lei Ren, Carmen Mota Reyes, Helmut Friess, Ihsan Ekin Demir

**Affiliations:** 1 Department of Surgery, Klinikum rechts der Isar, School of Medicine, Technical University of Munich, Ismaninger Str. 22, D-81675 Munich, Germany; 2 Department of General Surgery (Gastrointestinal Surgery), The Affiliated Hospital of Southwest Medical University, Luzhou, Sichuan China; 3 German Cancer Consortium (DKTK), Partner Site Munich, Munich, Germany; 4 CRC 1321 Modelling and Targeting Pancreatic Cancer, Munich, Germany; 5 Department of General Surgery, HPB Unit, School of Medicine, Acibadem Mehmet Ali Aydinlar University, Istanbul, Turkey

**Keywords:** Pancreatic cancer, Neoadjuvant therapy, Arterial resection, Immunotherapy

## Abstract

**Background:**

Neoadjuvant therapies (neoTx) have revolutionized the treatment of borderline resectable (BR) and locally advanced (LA) pancreatic cancer (PCa) by significantly increasing the rate of R0 resections, which remains the only curative strategy for these patients. However, there is still room for improvement of neoTx in PCa.

**Purpose:**

Here, we aimed to critically analyze the benefits of neoTx in LA and BR PCa and its potential use on patients with resectable PCa. We also explored the feasibility of arterial resection (AR) to increase surgical radicality and the incorporation of immunotherapy to optimize neoadjuvant approaches in PCa.

**Conclusion:**

For early stage, i.e., resectable, PCa, there is not enough scientific evidence for routinely recommending neoTx. For LA and BR PCa, optimization of neoadjuvant therapy necessitates more sophisticated complex surgical resections, machine learning and radiomic approaches, integration of immunotherapy due to the high antigen load, standardized histopathological assessment, and improved multidisciplinary communication.

## Introduction

**Fig. 1:**
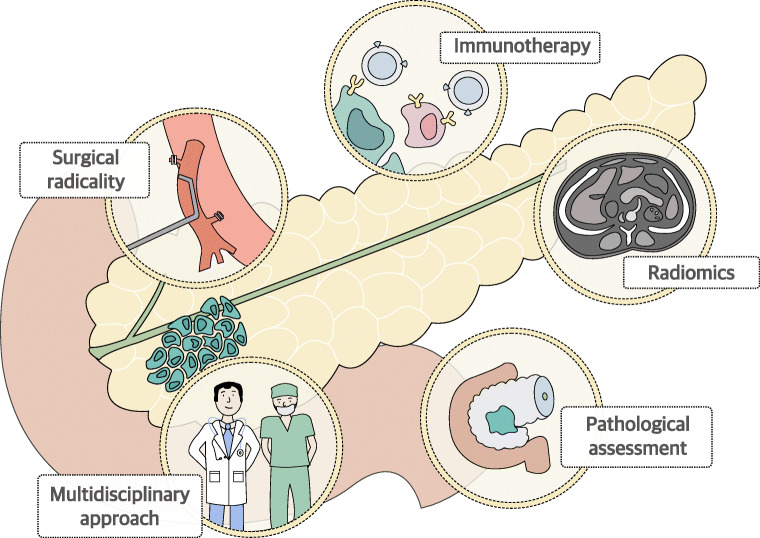
The evolving arms of a pleiotropic neoadjuvant therapy (neoTx) for locally advanced pancreatic cancer (PCa). We envision that the survival benefit through neoTx in locally advanced or borderline resectable PCa can be further improved via (1) more sophisticated approaches for complex surgical resections, (2) increased integration of radiomic approaches to staging and restaging after neoTx, (3) testing of immunotherapy in the neoadjuvant setting due to the relatively higher antigen load of the tumor, (4) worldwide standardization of the histopathological assessment, and (5) improved communication between all disciplines

The introduction of neoTx has led to a remarkable increase in the rate of surgical resections in PCa patients with LA or BR tumors, which were initially deemed inoperable at the time of diagnosis. However, two-thirds of these patients will develop local recurrences shortly after the operation [1]. In order to avoid disease relapse, surgeons have struggle to find ways to maximize R0 resections that still remain the only curative alternative for long-term survival in PCa. Although the first attempts of arterial resections (AR) in advanced tumors did not show the expected success, improved perioperative management and the integration of neoTx into multimodal therapy approaches have resulted in significantly reduced perioperative mortality and have proven the safety and feasibility of these radical approaches. Although neoTx is the standard of care for BR and LA tumors, its application on upfront resectable patients in order to downstage tumors and to increase surgical radicality is still subject of investigation. Furthermore, the introduction of immunotherapy to reactivate the pancreatic tumor microenvironment (TME) specially in neadjuvant settings constitutes a promising strategy for future multimodality PCa treatments (Fig. 1) [2, 3].

## NeoTx in borderline resectable and locally advanced PCa

Upfront surgery in BR and LA tumors has not elicited the expected survival benefit and is associated with high morbidity, low R0 resection rat, and high early-systemic recurrences [4]. The introduction of neoadjuvant approaches enabling the tumor downstaging has led to successful surgical resection in up to 60% of these patients [5]. However, neoTx not only decreases tumor size and facilitates surgical resection but also enables the selection of patients with a favorable tumor biology, who will benefit from radical resections [6]. A multicenter phase III randomized controlled trial (RCT) validated the use of neoTx in BR PCa patients. The initial analysis showed that neoTx resulted in increased R0 resection rates and prolonged disease-free survival (DFS) [7]. However, the final results showed that the neoTx protocol (preoperative gemcitabine-based chemotherapy combined with 15× 2.4 Gy radiotherapy) did not improve the overall survival [7]. After neoTx, PCa patients with LA tumors demonstrate favorable histopathological features with higher R0 resection rates and decreased frequency of lymph node metastasis and perineural and lymphatic vessel invasion [8]. These encouraging results have led to an increasing number of neoadjuvantly treated patients; however, not all of these patients ultimately undergo surgical exploration. Mellon et al. reported that 46 of 110 patients with BR PCa became unresectable due to local/distant progression or due to poor performance status that precluded resection after neoTx [9]. Importantly, therapeutic response to neoTx is not reliably reflected by the current imaging techniques. This highlights the need for multidisciplinary communications between surgeons and oncologists to ensure an unbiased selection of patients for surgical exploration and an optimized patient management in PCa.

Conventional computed tomography (CT), the most commonly used imaging modality for the initial determination of tumor stage and resectability of PCa, has striking limitations in the evaluation of vessel involvement after neoTx [10]. The recent introduction of whole-tumor radiomic analysis has opened a range of possibilities to assess therapy response and resectability in PCa in a quantitative and non-invasive manner. A supervised machine learning algorithm from diffusion-weighted magnetic resonance imaging allowed overall survival (OS) prediction with a high diagnostic accuracy as well as histopathological sub-stratification of PCa patients [11]. Recent reports also pointed out that the combination of radiomic features such as reduced tumor stiffness in endosonographic elastography or reduced intensity on PET-CT is able to assess therapy response in PCa after neoTx [12]. While still in need of validation studies, the large-scale implementation of such tools has the potential to revolutionize image interterpretation and individualized patient care [11]*.*

## NeoTx in resectable PCa: illusion versus reality

Although upfront surgery followed by adjuvant chemotherapy is still the recommendation for resectable PCa, this treatment fails to discriminate patients with undetected metastatic dissemination or aggressive tumor biology that may not benefit from surgical resection [13]*.* Furthermore, due to the high postoperative morbidity associated with pancreatic resections, up to 30% fail to receive or complete adjuvant chemotherapy [14]. The success of neoTx in BR/LA tumors has raised the question whether neoTx can improve prognosis in resectable patients, and clinical trials addressing to this matter are increasingly emerging.

The potential risk for patients with resectable PCa to develop local or distant tumor progression during neoTx, which might not have occurred in the setting of upfront resection, has been a recurrent argument against the use of neoTx in resectable tumors. The therapeutic paradigm of PCa is constantly evolving, and the focus has now turned toward the ability of the surgeon to remove the tumor radically. In this regard, neoTx may reduce surgical complexity by reducing the tumor bulk, the proportion of viable tumor cells, and the involvement of nearby vascular structures, resulting in an increased R0 resection rate [15].

Two early studies comparing the efficacy of gemcitabine-based neoTx with upfront surgery for resectable PCa determined its safety and feasibility but were terminated early due to slow recruitment and did not achieve statistically significant results [6, 16, 17]. Accordingly, two RCTs reported recently that neoTx is safe and effective without increased risk of surgical complications and was associated with favorable R0 resection rates in patients with resectable PCa [18]. In a large retrospective study, Mockdad et al. described prolonged survival in neoadjuvant-treated patients with early-stage PCa compared with upfront resected patients and thus provided further support for the use of neoTx as a patient selection tool in the management of resectable PCa [14]. Moreover, grade 3/4 toxicity in resectable PCa patients treated with neoTx was lower than in patients with BR/LA disease [19, 20]. In contrast, the recently published PREOPANC trial failed to any benefit in overall survival of patients with borderline or upfront resectable PCa (16.0 months with preoperative chemoradiotherapy versus 14.3 months with upfront surgery *P* = .096). Therefore, for early-stage, i.e., resectable, PCa, there is not enough scientific evidence for routinely recommending neoTx [7]. NeoTx in resectable PCa remains area of controversy and awaits the results of ongoing RCTs [21].

### Radical resection in PCa: “the holy grail”

Curative R0 resection remains the only chance for long-term survival in PCa [22, 23]; however, approximately half of the resections are microscopically incomplete and two-thirds of initially R0-diagnosed patients will develop local recurrence [24]. Despite the prognostic relevance of the pathological resection rate, a standard definition for R0 resection is still lacking, which leads to high variability on R0 resection rates that range between 15% and 92% [1, 24–26]. After the introduction of a standardized pathology protocol consisting of axial slicing technique, multicolor margin staining and extended sampling, and a circumferential resection margin (CRM) > 1 mm, the R1 rate significantly increased from 14 to 76% in a retrospective study carried out by Esposito et al. [27, 28]. These observations indicate that resection margin involvement is a common finding in PCa which is often underestimated due to the lack of a standardized pathological examination of all relevant margins [28] and insinuated the need to increase surgical radicality in other to obtain wider resection margins and higher R0 rates. In line with these results, a retrospective study with 360 patients revealed similar local recurrence rates of R0- and R1-staged PCa patients suggesting the widespread presence of undiagnosed microscopic residual disease. Further intercontinental discrepancy is reported on the definition of R0 status, which is 0-mm tumor distance from resection margin in the USA and > 1 mm in Europe and Australia [1, 24, 27, 29, 30]. In our recent meta-analysis assessing the importance of the resection status in PCa, we demonstrated that even with standardized pathology protocols, resection margin’s prognostic validity may be primarily confined to pancreatic head tumors [24].

Pancreatic surgeons are continuously developing new strategies to increase surgical radicality and improve R0 resection rates [4]. The feasibility of portomesenteric venous resection has been widely demonstrated. In contrast, extended arterial involvement remains a controversial issue in the management of PCa. Although tumor encasement of the superior mesenteric artery, common hepatic artery, or celiac artery defines local irresectability according to current guidelines, advances in the field of pancreatic surgery have turned the focus on redefining strategies that allow more radical approaches involving the resection and reconstruction of major peripancreatic arteries, to achieve R0 resection in patients without distant metastasis [4, 31].

In the first meta-analysis evaluating AR in patients undergoing pancreatectomy for PCa, AR was discouraged as standard of care and was associated with remarkably higher perioperative morbidity (OR = 2.17) and mortality (OR = 5.04) and poor survival (OR = 0.50) [4]. Conversely, in a recent study, Del Chiaro et al. demonstrated the feasibility and safety of AR in pancreatectomy, which was accompanied by increased survival compared with palliative procedures and showed no difference in postoperative mortality and morbidity, even though it was associated with longer operation time and higher blood loss [31, 32]. Consistent with these results, Sonohara et al. demonstrated that PCa patients with AR had marginally higher recurrence-free survival and longer overall survival without a significant increase in the incidence of severe postoperative complications [33]. Current studies evaluating celiac artery resection also showed that these procedures can be performed safely and with an encouraging median survival [32, 34]. Further analyses suggested the improvement to be a consequence of newly developed and more effective chemotherapeutical regimens used in neoadjuvant settings. The increasing use of neoTx has notably increased the rate of R0 resections in patients with initially suspected arterial infiltration [35] and has led to significantly higher survival rates (78.8%) compared with patients who underwent upfront surgery (26.7%) [36]. In line with these results, Bachellier et al. reported remarkably prolonged survival in neoadjuvantly treated patients (23 months) compared with upfront resected PCa patients (13.7 months) after extended pancreatectomies involving AR [37]. Therefore, neoTx appears to provide an additional benefit to AR in patients with BR and LA PCa undergoing extended pancreatectomy by decreasing tumor burden and arterial invasion [33, 38]. In the case of adequate therapeutic response and good performance status, resectability should be re-assesed via surgical exploration, as cross-sectional images often fail to identify the extent of the remaining viable tumor. Combining AR with pancreatectomy in these cases increases the feasibility of R0 resection, which is still the only option to achieve long-term survival [39]. Here, neoTx should be performed rather than upfront surgery. Clinical trials analyzing the superiority of combined chemotherapeutical regimes and radical surgical resections are still needed and ongoing [4, 40–42].

### Immunotherapy as a novel neoadjuvant approach in PCa

Cancer immunotherapy has demonstrated remarkable therapeutic efficacy in many solid malignancies [43]. Due to low tumor mutational burden and the presence of a highly immunosuppressive TME, immunotherapies have consistently failed to elicit the expected outcomes in PCa [44]. This limitation may be circumvented by the application of immunotherapy in a neoadjuvant setting, with the primary tumor serving as an antigen source for in situ T cell priming that may unleash a more potent antitumoral immune response compared with adjuvant approaches [45]. Current neoTx in PCa mostly relies on classical chemotherapy regimens such as FOLFIRINOX and does not make use of immune-based and molecular-targeted therapies. Surprisingly, we observed an immunological shift toward more cytotoxic inflammation in the TME of PCa after conventional neoTx. This was mainly due to the depletion of immunosuppressive cells like regulatory T cells (Treg cells) [46] and myeloid-derived suppressor cells (MDSCs) [45, 47]. These results suggested that neoTx is able to prime the TME and potentiate the effect of immunotherapy by boosting the local antitumor immune response in PCa.

Ongoing trials on PCa are now focusing on combinatorial approaches exploiting the ability of cancer vaccines to promote T cell recruitment followed by the subsequent activation of cytotoxic cells by checkpoint inhibitors (ICIs) or immunomodulatory agents [48]. The inhibition of T cell checkpoints such as T lymphocyte protein 4 (CTLA4) and programmed cell death protein 1 (PD-1) has shown enormous promise in a number of cancer types [49, 50] by unleashing tumor-specific cytotoxic T cells that already reside in TME before treatment [51]. So far, none of these antagonists has proven effective in PCa [48]. However, the combination of a CD40 agonist with nab-paclitaxel plus gemcitabine resulted in partial response in 4 of 21 patients with PCa, and a clinical trial for its use as a neoadjuvant is underway (NCT02588443). Adoptive immunotherapy involves the injection of tumor reactive immune cells into patients and has increasingly gained attention over the past years. Although the first clinical trials with chimeric antigen receptor (CAR) T cells or tumor-pulsed dendritic cells in advanced PCa have shown promise [48], adoptive approaches have yet not been tested in neoadjuvant settings in PCa. The number of clinical trials evaluating the use of neoadjuvant immunotherapy is limited compared with its use within palliative approaches (Table 1).

**Table 1:** Ongoing clinical trials evaluating the effect of immunotherapy in PCa

NCT identifier	Phase	Allo-cation	Arms	Target accrual	Primary endpoint	Recruitment status	Projected completion date	Disease status	neoTx
NCT03114631	III	Non-R	DCs pulsed with tumor lysate; DCs pulsed with MUC-1/WT-1 peptides; no intervention	30	PR or CR at 1 year	Completed	May 19	LA/M	no
NCT03989310	III	N/A	Manganese chloride; nab-paclitaxel, gemcitabine; anti-PD-1 antibody	20	AEs and DCR	Recruiting	Mar 21	LA/M	no
NCT03323944	I	Non-R	huCART-meso cells	18	AEs	Recruiting	Sep 21	NR/M	no
NCT03008304	III	R	High-activity NK; no intervention	20	RECIST	Completed	Dec 19	M	no
NCT03165591	III	N/A	V3-P	30	Tumor burden, CA19.9	Recruiting	Dec 20	NR/M	no
NCT03180437	III	R	IRE surgery; IRE plus γδ T cells	60	PFS, OS	Completed	43,617	LA	no
NCT03329248	III	N/A	ALT-803; ETBX-011; GI-4000; naNK; avelumab; bevacizumab; capecitabine, cyclophosphamide; fluorouracil; leucovorin, nab-paclitaxel; iovaza, oxaliplatin, SBRT	80	RECIST, AEs	Completed	Dec 19	Progress after SoC	no
NCT02718859	III	R	NK cells; IRE	60	PFS, OS	Completed	Mar 19	NR/M	no
NCT03193190	III	R	Nab-paclitaxel, gemcitabine, atezolizumab, selicrelumab, AB928, tiragolumab, cobimetib, PEGPH20, BL8040, RO6874281	260	RECIST, AEs	Recruiting	Nov 21	NR/M, progess after SoC	no
NCT02261714	III	N/A	TG01	32	DTH responses and proliferative T cell responses	Completed	May 19	ATx	no
NCT03941457	I/II	N/A	BiCAR-NK cells (ROBO1 CAR-NK cells)	9	CTCAE	Active, not recruiting	May 20	M	no
NCT03168139	I/II	N/A	Olapteselpegol + pembrolizumab + combination therapy	20	AEs, ECG, vital signs	Completed	Mar 20	M	no
NCT03153410	I	N/A	Cyclophosphamide, GVAX, pembrolizumab, IMC-CS4	12	OS, DFS, ORR, RECIST, resectability, pRR, PFS	Active, not recruiting	Sep 20	BR	yes
NCT03816358	I/II	Non-R	Anetumab ravtansine, gemcitabine, ipilimumab, nivolumab	64	MTD	Recruiting	Apr 21	NR/M	no
NCT04050085	I	N/A	Nivolumab, radiation Tx, TLR9 agonist SD-101	6	AE, clinical laboratory	Recruiting	Aug 20	M, Progress after SoC	no
NCT03373188	I	R	Anti-SEMA4D monoclonal antibody, VX15/2503, ipilimumab, nivolumab	32	T cell infiltration	Recruiting	Dec 22	Re	yes
NCT03970252	I/II	N/A	Fluorouracil, irinotecan, leucovorin, nivolumab, oxaliplatin	36	Pancreatic fistula, pCR	Recruiting	Apr 22	BR	yes
NCT03252808	I	R	TBI-1401 (HF10), gemcitabine, nab-paclitaxel, TS-1	36	AEs, ORR, RECIST, PFS	Active, not recruiting	Mar 35	NR/M	no
NCT03269526	I/II	N/A	EGFR BATs	22	AEs, OS	Recruiting	Mar 23	NR/M	no
NCT03767582	I/II	R	SBRT	30	CTCAE, immune response	Recruiting	Mar 22	NR	no
NCT03745326	I/II	Non-R	Cyclophosphamide, fludarabine, aldesleukin, anti-KRAS G12D mTCR PBL	70	AEs, response rate	Recruiting	Dec 28	NR/M	no
NCT03953235	I/II	Non-R	GRT-C903, GRT-R904, nivolumab, ipilimumab	144	AEs, SAEs, DLT, ORR, RECIST, RP2D	Recruiting	Dec 23	NR/M	no
NCT01351103	I	Non-R	LGK974, PDR001	184	MTD, RDE	Recruiting	Mar 22	NR/M, progress after SoC	no
NCT03058289	I/II	Non-R	INT230-6, anti-PD-1 antibody, anti-CTLA-4 antibody	110	CTCAE	Recruiting	Oct 22	Progress after SoC	no
NCT03611556	I/II	R	Oleclumab, durvalumab, gemcitabine, nab-paclitaxel, oxaliplatin, leucovorin, 5-FU	339	AEs, ORR, RECIST, ECG, clinical laboratory	Active, not recruiting	Dec 21	M	no
NCT03336216	II	R	Cabiralizumab, nab-paclitaxel, onivyde, nivolumab, fluoruracil, gemcitabine, oxaliplatin, leucovorin, irinotecan	179	PFS, RECIST	Active, not recruiting	Dec 20	NR/M, progress after SoC	no
NCT02907099	II	N/A	CXCR4 antagonist BL-8040, pembrolizumab	23	ORR, RECIST	Active, not recruiting	Dec 22	Progress after SoC	no
NCT03161379	II	N/A	Clyclophosphamide, nivolumab, GVAX, SBRT	50	pCR	Recruiting	Jan 23	BR	yes
NCT03723915	II	N/A	Pembrolizumab, wild-type reovirus	30	ORR, RECIST	Active, not recruiting	Jun 21	NR/M, progress after SoC	no
NCT02305186	I/II	R	Pembrolizumab, NeoCRTx	56	Number of TILs, DLT	Active, not recruiting	Dec 22	Re/BR	yes
NCT03563248	II	R	FOLFIRINOX, losartan, nivolumab, SBRT	160	R0-resection rate	Recruiting	Dec 25	BR/LA	yes
NCT01088789	II	R	PANC 10.05 pcDNA-1/GM-Neo and PANC 6.03 pcDNA-1 neo vaccine.	72	DFS, CTCAE	Recruiting	Apr 23	Re/BR	yes
NCT03190265	II	R	Cyclophosphamide, nivolumab, ipilimumab, GVAX pancreas vaccine, CRS-207	63	ORR, RECIST	Recruiting	Oct 23	M	no
NCT03006302	II	R	Epacadostat, pembrolizumab, CRS-207, CY, GVAX	70	Recommended dose, 6-months survival	Recruiting	Jun 23	NR/M, progress after SoC	no
NCT01896869	II	R	Ipilimumab, vaccine, FOLFIRINOX	83	OS	Completed	May 19	M	no
NCT03250273	II	Non-R	Entinostat, nivolumab	54	ORR, RECIST	Recruiting	Nov 20	NR/M	no
NCT03717298	II	N/A	Ocoxin-Viusid®	30	EORTC QLQ-C30,	Recruiting	Dec 20	NR/M	no
NCT03767582	I/II	R	SBRT, nivolumab, CCR2/CCR5 dual antagonist, GVAX	30	CTCAE, immune response	Recruiting	Mar 22	LA	yes
NCT02446093	II	R	GMCI, CTx, radiation, surgery	38	Resection rate, CTC	Recruiting	Dec 22	BR/LA	yes
NCT03727880	II	R	Pembrolizumab, defactinib	36	pCR	Recruiting	May 23	Re	yes
NCT03806309	II	R	FOLFIRI, OSE2102, nivolumab	156	OS	Recruiting	Dec 23	LA/M	no
NCT03977272	III	R	Combination drug, CTx	110	OS	Recruiting	Mar 22	M	no
NCT03983057	III	R	Anti-PD-1 monoclinal antibody	830	PFS	Recruiting	Apr 22	BR/LA	yes

*R* randomized, *Non-R* non randomized, *LA* locally advanced, *NR* not resectable, *M* metastatic, *Re* resectable, *PR* partial response, *CR* complete response, *pCR* pathological complete response, *pRR* pathological response rate, *DCR* disease control rate, *AE* adverse events, *CAR T cells* chimeric antigen receptor modified T cells, *IRE* irreversible electroporation, *PFS* progression-free survival, *OS* overall survival, *RECIST* response evaluation criteria in solid tumors, *SoC* standard of care, *CTx* chemotherapy, *ATx* adjuvant therapy, *DHT* delayed hypersensibility, *CTCAE* common terminology criteria for adverse events, *ECG* electrocardiogram, *PFS* progression-free survival, *MTD* maximum tolerated dose, *STBR* stereotactic body radiation, *ORR* objective response rate, *RP2D* recommended phase 2 dose, *RDE* recommended dose for expansion, *DCR* disease control rate, *TIL* tumor-infiltrating lymphocytes, *DLT* dose-limiting toxicity

In low mutational tumors such as PCa, neoTx may be particularly beneficial to potentiate the antitumor immune response compared with adjuvant approaches, as the tumor epithelium itself remains an essential source for the release of tumor antigens and cross-priming of tumor-directed T cell responses. This important reservoir for induction of tumor-directed immune responses is no longer available after tumor resection [52]**.** Liu et al. administrated various combination immunotherapies in either neoadjuvant or adjuvant setting and discovered that regardless of the type of immunotherapy used, neoadjuvant approaches were superior to adjuvant treatments in primary breast tumors [53]. In line with these observations, Brooks et al. demonstrated that only the combination of neoadjuvantly applied gemcitabine and a PD-1 inhibitor, but not adjuvant treatment, effectively suppressed local tumor recurrence and improved survival in a transgenic mouse model of PCa [52].

## Conclusion

NeoTx leads to an immunologic shift toward a more effective antitumor immune response in the pancreatic TME, which recently provided impetus for studying the possibility of combining neoTx with immunotherapy in patients with PCa. Furthermore, neoTx leads to increased R0 resection rates and reduces the complexity of pancreatic surgical resections in LA/BR PCa patients. After neoTx, the postoperative morbidity associated with AR in pancreatectomy was similar to less radical approaches, leading the way to more sophisticated and radical surgical strategies in PCa. However, for resectable PCa, the overall survival benefit through neoTx does not exist in a convincing extent. Optimal drug regimens, timing of surgery with regard to therapy, and the role of additional immunotherapy still need to be defined. Balancing the optimal therapy for PCa will be complex and will require correct patient stratification, the use of combination strategies, and improved interdisciplinary cooperation.
